# Clinical AI is Not (Yet) Trustworthy-But It Could Be

**DOI:** 10.2196/85433

**Published:** 2026-04-29

**Authors:** Ali Saad, Sofia B Dias, Ghada Alhussein, David Lyreskog, Ioannis Gerasimou, Beatriz Alves, Μaarten de Vos, Ioannis Drivas, John Zaras, Andreas Stergioulas, Iskanter Bensenousi, Leontios Hadjileontiadis, Christos Chatzichristos, Stelios Hadjidimitriou

**Affiliations:** 1 AINIGMA Technologies Leuven Belgium; 2 Faculdade de Motricidade Humana Universidade de Lisboa Centro Interdisciplinar de Estudo da Performance Humana Lisbon Portugal; 3 Department of Biomedical Engineering and Biotechnology College of Medicine and Health Sciences Khalifa University of Science and Technology Abu Dhabi United Arab Emirates; 4 NEUROSEC Department of Psychiatry University of Oxford Oxford United Kingdom; 5 Department of Electrical and Computer Engineering School of Engineering Aristotle University of Thessaloniki Thessaloniki Greece; 6 Faculdade de Motricidade Humana University of Lisbon Lisbon Portugal; 7 STADIUS Center for Dynamical Systems, Signal Processing, and Data Analytics Department of Electrical Engineering KU Leuven Leuven Belgium; 8 Diadikasia Business Consulting Symvouloi Epicheiriseon AE Athens Greece; 9 Squaredev Brussels Belgium; 10 Information Technologies Institute Centre for Research and Technology Hellas Thessaloniki Greece

**Keywords:** trustworthy artificial intelligence (AI), clinical AI, ALTAI Framework, assessment list for trustworthy artificial intelligence, AI-PROGNOSIS European research initiative, lifecycle safeguards, ethical AI integration

## Abstract

The growing emphasis on trustworthy artificial intelligence (AI) in health care reflects a shift away from models optimized for predictive performance toward governable and auditable systems that can be adopted and sustained in clinical practice. Nonetheless, many clinical AI applications continue to privilege technical performance while underaddressing ethical, regulatory, and societal considerations, leading to concerns around robustness, transparency, and clinical adoption. To address this, governance frameworks such as the Assessment List for Trustworthy Artificial Intelligence (ALTAI) have been proposed to operationalize trust-related requirements across the AI lifecycle. However, evidence on the practical use of these frameworks remains limited. In this Viewpoint, we describe the application of ALTAI as a procedural governance framework within the Horizon Europe AI-PROGNOSIS project, which aims to support Parkinson disease diagnosis and care through predictive models and digital biomarkers derived from everyday devices. The seven ALTAI requirements (ie, human agency and oversight; technical robustness and safety; privacy and data governance; transparency; diversity, nondiscrimination, and fairness; societal and environmental well-being; and accountability) were mapped to key stages of the AI lifecycle within the project, including design and specification, data preparation, model development and validation, user interface and user experience deployment, external prospective validation, and overarching management and workflow. To examine how these requirements were perceived in practice, we conducted a structured internal survey among AI developers and data scientists involved in the AI-PROGNOSIS project (n=10). Participants rated the relevance of the 17 ALTAI subdomains using a three-point prioritization scale. Technical accuracy, data governance, and privacy were consistently rated as highly relevant, whereas societal impact received the lowest prioritization. This pattern reflects a documented tension in AI development, where technical teams tend to deprioritize broader societal concerns under delivery and performance constraints. Nonetheless, this work should be interpreted as a context-specific case study rather than a validation of ALTAI. The small sample size and project-specific setting limit generalizability, and these findings should not be considered as representative of broader clinical AI development. Overall, by making prioritization gaps explicit and embedding multidisciplinary review across lifecycle checkpoints, this case study illustrates how structured governance frameworks can surface implementation tensions and support accountable AI development. While these approaches do not resolve all of the aforementioned challenges, they provide practical guidance for integrating trust-related considerations into clinical AI projects.

## Introduction

Artificial intelligence (AI) has seen accelerating development and adoption across health care domains, driven by advances in machine learning (ML), data availability, and computational power [1]. While this technological momentum has led to significant gains in diagnostic accuracy, prognostic modeling, and treatment optimization, the integration of AI systems into routine health care remains cautious and uneven. This reticence, while partially attributable to regulatory inertia and data access limitations, is fundamentally rooted in a deeper concern: the perceived trustworthiness of AI-driven systems [2,3]. This is, in fact, a critical challenge, as performance alone is insufficient without ensuring reliability, ethical alignment, and public trust [4], especially as health care AI systems transition from proof of concept to clinical deployment.

Trust in clinical AI transcends conventional performance metrics. It is not reducible to algorithmic accuracy or validation statistics alone, but rather represents a composite property that encompasses transparency, interpretability, accountability, and alignment with both clinical values and ethical principles [5,6]. Recent works have highlighted how the implementation of AI in health care is increasingly shaped not only by technical feasibility, but by the complex interplay of governance, institutional norms, and frontline practices [7,8]. Trustworthiness in AI is increasingly recognized as a multidimensional construct that encompasses not only compliance with technical benchmarks, but also alignment with ethical principles and regulatory standards [9]. Such an alignment must extend beyond end-product validation to permeate the entire lifecycle of system development. Procedural approaches, those that embed trust-oriented safeguards from design to deployment, are essential to achieving this [10]. From the Viewpoint of end users, including clinicians, patients, and institutions, trust is not simply earned by retrospective audit or certification; instead, it is cultivated over time, shaped by system behavior, user experience (UX), and organizational context [11].

Despite the proliferation of frameworks and normative guidance, ranging from high-level ethical principles to emerging regulatory instruments, a persistent implementation gap remains [11,12]. Existing instruments frequently emphasize outcomes rather than mechanisms; they evaluate trust post hoc, rather than embedding it procedurally throughout the AI lifecycle. This disconnect highlights the absence of a pragmatic scaffolding to guide trustworthy AI design and deployment within high-stakes environments, such as health care.

This Viewpoint advances a procedural approach to trustworthiness in clinical AI, drawing upon the Assessment List for Trustworthy Artificial Intelligence (ALTAI) developed by the High-Level Expert Group on AI [13], as a representative tool for operationalizing ethical and regulatory principles. Unlike purely aspirational codes, the ALTAI framework offers a practical, step-by-step checklist that can be directly integrated into project workflows. It delivers concrete guidance throughout the entire AI lifecycle, including design specification, data governance, model development, evaluation, and deployment, and includes defined metrics and procedures to promote compliance and transparency [13]. To contextualize this approach, we examine the AI-PROGNOSIS project [14], a European initiative focused on the development of predictive models for Parkinson disease (PD), as a case study. AI-PROGNOSIS was selected as the case study for applying the ALTAI framework because it concentrates on several recurrent challenges in clinical AI. The project aims to generate individualized PD risk scores, PD progression forecasts, and medication response estimates from multimodal health data. This requires analyzing data from heterogeneous data sources and longitudinal modelling, aiming, for instance, to provide support for therapeutic decision-making. These characteristics make AI-PROGNOSIS a suitable and demanding test case for examining how ALTAI can guide trustworthiness, transparency, and risk mitigation in complex clinical AI systems beyond this specific consortium, illustrating how a procedural framework can shape real-world system design.

By anchoring trustworthiness in procedural steps rather than retrospective assessments, this Viewpoint contributes to a growing body of literature calling for actionable strategies to embed ethical and regulatory principles into real-world AI systems [7,8]. In the sections that follow, we articulate the conceptual rationale for procedural trust, outline the ALTAI framework, illustrate its instantiation across the AI development continuum via the AI-PROGNOSIS case study, and reflect on the practical, ethical, and sociotechnical tensions encountered during implementation in health care innovation.

### From Principles to Procedure: Why Trust in AI Needs a Blueprint

The proliferation of ethical guidelines for AI has revealed a growing consensus: trust is essential for the responsible deployment of AI in health care. Yet, despite the emergence of high-level principles, such as fairness, transparency, and accountability, there remains a persistent gap between normative aspirations and practical implementation [15,16]. This disconnect has prompted calls for procedural frameworks that can translate abstract values into actionable design and governance strategies [17]. This approach has several benefits over traditional principle-based frameworks of trust, which typically rely on specific targets and retrospective evaluations.

Nevertheless, trust in AI is neither a monolithic concept nor an intrinsic property of the system; rather, it is a system-level outcome shaped by dynamic interactions among users, institutions, and broader sociotechnical environments [18]. It emerges from the interplay of interrelated technical and ethical dimensions, such as explainability, robustness, and fairness, none of which are sufficient in isolation. Cultivating trust, therefore, entails iterative, lifecycle-spanning processes that embed normative safeguards into the design, development, deployment, and governance of AI systems [19,20].

In alignment with the ALTAI framework [13] and International Organization for Standardization/International Electrotechnical Commission Technical Specification (ISO/IEC TS) 5723:2022 [21], we identify eight core dimensions, that is, robustness, generalization, explainability, accountability, transparency, reproducibility, fairness, and privacy, as foundational to Trustworthy AI in health care. These dimensions reflect a synthesis of normative principles and practical requirements for clinical-grade AI systems. In particular, robustness refers to a system’s capacity to perform reliably under uncertainty, such as noisy inputs, adversarial perturbations, or incomplete records, without significant loss of function [9]. However, robustness must coexist with usability and interpretability, particularly in clinical environments. Closely related is generalization, the model’s ability to extrapolate to unseen data, which remains a fundamental challenge given the risk of underfitting or overfitting, especially in small or biased datasets [19,22,23].

Explainability is a context-sensitive construct that varies across stakeholders, clinicians, patients, regulators, and developers [20]. It may be achieved through post hoc methods (eg, Shapley additive explanations and local interpretable model-agnostic explanations) or interpretable models, each with trade-offs in fidelity and scalability [24]. Accountability demands clear traceability of decisions to responsible entities [25], while transparency, its enabling counterpart, requires open disclosure of model purpose, data provenance, and performance characteristics [26]. Achieving transparency often involves managing organizational or proprietary constraints. Reproducibility, a pillar of scientific integrity, remains elusive in ML due to nondeterminism in training processes and hardware dependencies [27]. Fairness, arguably the most socially charged dimension, seeks to mitigate bias introduced during data collection, model design, and deployment [9,28]. Technical responses span pre-, in-, and postprocessing interventions and must be informed by socioethical theories of discrimination and equity [22,28,29]. Finally, privacy safeguards not only identifiers but also individual autonomy over data use [12]. While techniques like differential privacy, de-identification, and data minimization offer protection, they may constrain model expressiveness or transparency [30]. These trade-offs underscore the necessity of procedural frameworks that treat trust not as a checklist but as a dynamic property shaped by ongoing design, validation, and governance.

While often treated as discrete targets, the aforementioned dimensions are deeply interwoven and must be addressed through integrated, context-sensitive design strategies across the AI lifecycle [23]. Attempts to enhance one dimension, such as increasing transparency, can inadvertently compromise another, such as protecting proprietary data or patient privacy. This interplay reinforces the need for procedural frameworks that consider trustworthiness as a system-wide property, developed iteratively and contextually throughout the AI lifecycle.

It is useful to clarify that the eight ISO/IEC TS 5723:2022 dimensions are introduced as foundational properties that define what constitutes a trustworthy clinical AI system, whereas the seven ALTAI requirements provide a procedural structure for how these properties can be operationalized across the AI lifecycle. Several of the ISO dimensions (eg, robustness, transparency, fairness, privacy, and accountability) map directly onto ALTAI’s requirements, while others (eg, generalization and reproducibility) are implicitly addressed through ALTAI’s emphasis on technical robustness, documentation, and oversight. This alignment positions ALTAI not as an alternative to the ISO dimensions, but as a practical mechanism for embedding them into development workflows, as discussed next.

### ALTAI as a Procedural Anchor

While core dimensions of Trustworthy AI, such as robustness, fairness, and transparency, provide conceptual structure, their realization in clinical settings requires systematic, context-sensitive implementation. From the Asilomar AI Principles [31] and the Montreal Declaration [32], to institutional efforts like AI4People [6] and the Organisation for Economic Co-operation and Development guidelines [33], much of this work has emphasized normative commitments, fairness, accountability, transparency, and safety. National strategies, including those from China [34], the United Kingdom [35], the United States [36], and the European Union [37], have begun translating these principles into policy and regulation. In the health care domain, oversight by bodies such as the US Food and Drug Administration [36] and the National Institute of Standards and Technology [38] adds additional complexity, particularly for high-risk systems. In particular, the US Food and Drug Administration guidance on AI and ML-enabled software as a medical device covers good ML practices, including data management, validation, and postmarket monitoring [36]. Similarly, the National Institute of Standards and Technology AI Risk Management Framework provides voluntary lifecycle risk management, emphasizing governance, mapping, measurement, and management of risks such as bias, robustness, and explainability in health care settings [38].

Among these efforts, the European Union’s (EU’s) ALTAI [39], developed by the high-level expert group on artificial intelligence in 2020 [33], stands out as a concrete procedural framework for embedding trust across the AI development lifecycle. Unlike principle-driven charters, ALTAI codifies seven actionable requirements: human agency and oversight, technical robustness and safety, privacy and data governance, transparency, diversity and fairness, societal well-being, and accountability. These dimensions are designed not as abstract endpoints, but as iterative checkpoints, aligning design practices with ethical and regulatory imperatives at each stage of AI development. Its web-based tool supports structured self-assessment and generates visual diagnostics, such as radar plots, summarizing strengths and deficiencies, thus enabling continuous monitoring, recommended next steps, and targeted refinement.

Albeit scoring details remain opaque, ALTAI represents one of the most widely adopted instruments for proceduralizing trust in AI workflows. To assess its translational value in applied clinical research, we adapted ALTAI within the AI-PROGNOSIS project. This adaptation aligned technical design efforts with structured trust requirements, providing a foundation for identifying risk points, embedding ethical safeguards, and supporting internal reflection among development teams. In the following section, we present this case study as an applied instantiation of ALTAI, illustrating how trust-oriented governance can be realized through procedural integration.

### Operationalizing Procedural Trust: The AI-PROGNOSIS Case Study

To evaluate the ALTAI procedural trust framework in clinical AI, we conducted a structured assessment within the AI-PROGNOSIS project, which aims to generate individualized PD risk scores, PD progression forecasts, and medication response estimates, using ML techniques applied to multimodal health data. AI‑PROGNOSIS was selected as the case study since it embodies several characteristics that typify the broader challenges of clinical AI. The project integrates multimodal health data, including clinical records, wearable‑derived digital biomarkers, and longitudinal assessments, and supports high‑stakes decisions related to PD. These features create a complex technical and ethical landscape in which issues of robustness, fairness, transparency, and data governance are particularly salient. As such, AI‑PROGNOSIS provides a demanding and representative context for stress‑testing how a procedural framework like ALTAI can be operationalized across the AI lifecycle.

A targeted survey (Table S1 in Multimedia Appendix 1) was administered to evaluate the relevance of ALTAI’s seven requirements in this context. The respondents included ten AI developers and data scientists across five partner institutions (two academic centers and three SMEs) of the AI-PROGNOSIS consortium. The group included eight male and two female participants, with a mean age of 40.2 (SD 8.9) years, and an average of 13.3 (SD 11) years of professional experience. All participants were directly involved in technical design or data pipeline development for the AI-PROGNOSIS platform.

The survey asked participants to rate the perceived importance of 19 ALTAI subgroups (Figure S1 in Multimedia Appendix 1), such as fallback plans, explainability, fairness, and environmental impact, using a three-point scale (low=1, medium=2, and high=3). When a subgroup was considered by a participant to be unrelated to the project, it was assigned a value of zero. The average importance rating was then calculated using only the non‑zero ratings, averaged across all subgroups and participants. To increase the resolution of the resulting average importance score, it was rescaled from 1-3 to the range of 1-10 by multiplying its value by a factor of 10/3.

The main aim was to identify which components were considered most critical for a health care-focused AI system in development. In fact, the survey was designed as an internal prioritization exercise rather than a statistical evaluation of ALTAI. The 10 respondents were members of the AI‑PROGNOSIS technical work packages and were selected because they were directly responsible for model development, data engineering, or pipeline integration within the project. In this context, roles such as “AI developer” and “data scientist” overlap substantially, and the survey did not aim to distinguish between them. The sample size reflects the bounded size of the consortium’s technical team, and no claims of statistical significance or generalizability are made. For this reason, descriptive characteristics are reported without inferential interpretation, and the survey is explicitly framed as capturing the perspectives of this specific project team rather than representing broader trends in clinical AI. Its purpose was to surface internal prioritization patterns across ALTAI subdomains to inform project‑specific design decisions, not to validate the ALTAI framework itself. From this perspective, potential for selection bias can be acknowledged, as all participants were selected precisely because they were directly involved in AI‑PROGNOSIS development, and their perspectives therefore reflect the priorities and constraints of this specific consortium rather than those of the broader clinical AI community.

**Figure 1:**
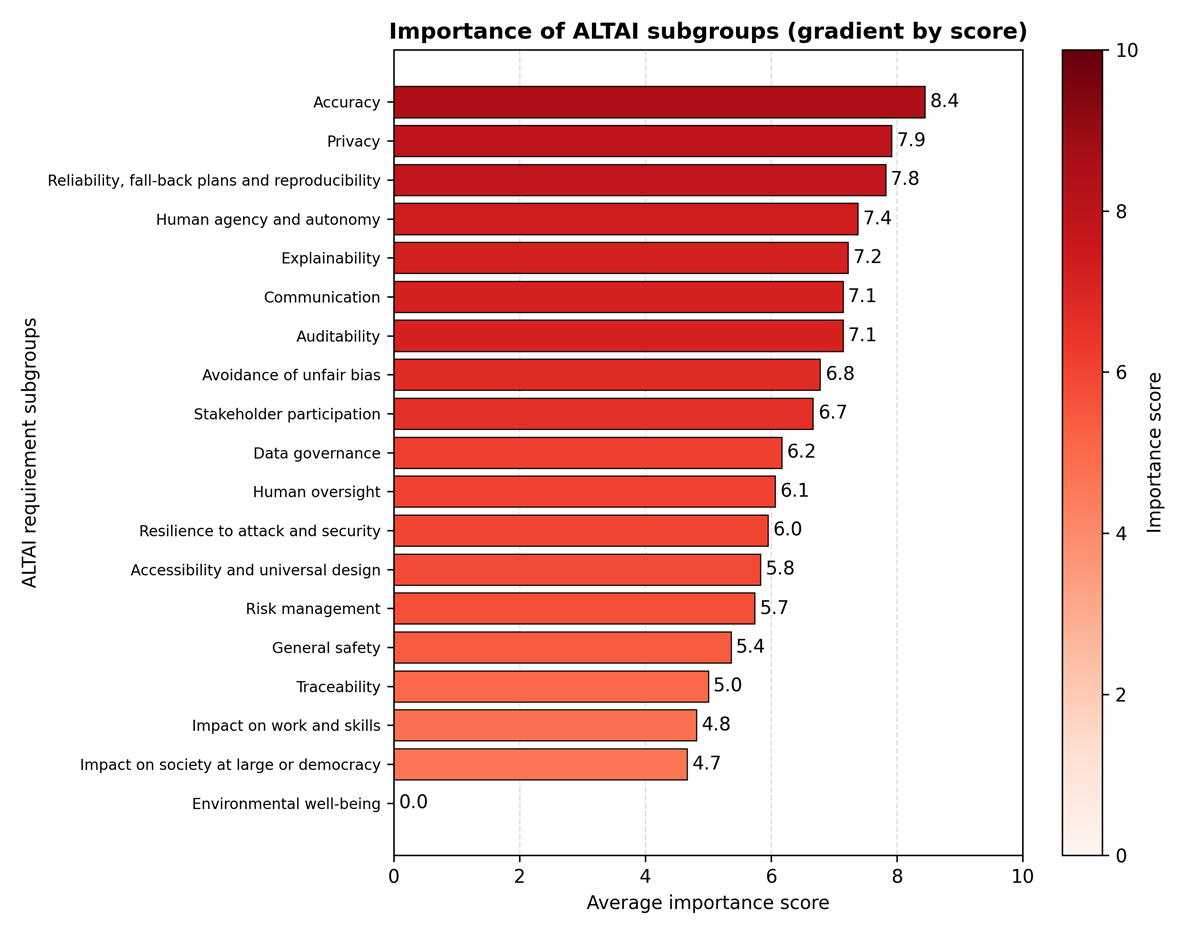
Average importance scores per requirement for ALTAI subgroups. Ratings were derived from a structured survey conducted with AI experts in the AI-PROGNOSIS project, reflecting the perceived relevance of each ALTAI requirement to clinical AI development. ALTAI: Assessment List for Trustworthy Artificial Intelligence, AI: Artificial Intelligence.

**Figure 2:**
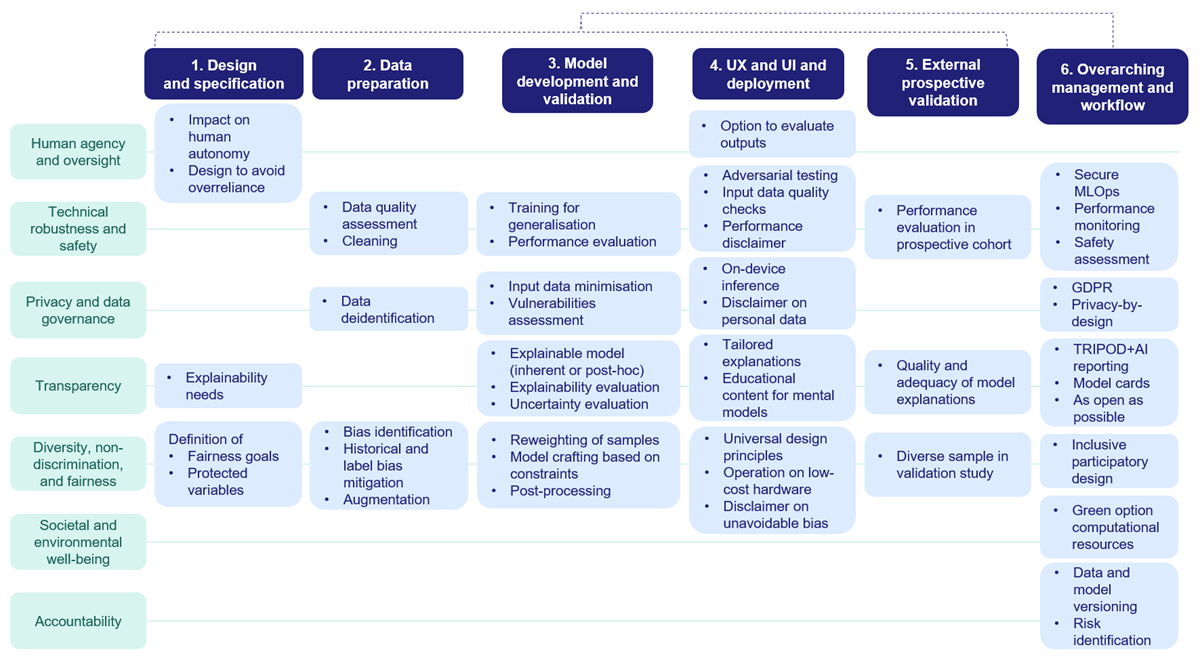
Trustworthy AI components embedded in the AI-PROGNOSIS framework. The graphical representation summarizes how dimensions such as explainability, user experience (UX), user interface (UI), and accountability are operationalized within the AI-PROGNOSIS project, supporting trust by design. AI: Artificial Intelligence.

Figure 1 illustrates the average importance ratings provided by AI experts across the seven ALTAI requirements. More specifically, subgroups related to accuracy, privacy, reliability, fallback planning, human agency and autonomy, explainability, communication, and auditability received the highest range of average importance score (8.4-7.1), respectively. Bias mitigation, stakeholder participation, data governance, human oversight, and resilience to attack and security received an average importance score within the range of 6.8-6.0, respectively. The third range of 5.8-5.0 included accessibility and universal design, risk management, general safety, and traceability, respectively. In contrast, requirements addressing the impact on work and skills, along with the impact on society at large or democracy, were rated as less critical with average importance scores of 4.8 and 4.7, respectively. Finally, the environmental well-being was considered as not related to the project and resulted in zero. In addition, Figure S1 in Multimedia Appendix 1 offers a more granular view by displaying the average importance scores for each ALTAI subgroup. Table S2 in Multimedia Appendix 1 provides the full set of ALTAI checklist responses alongside the corresponding system-generated recommendations. Moreover, to support integration across the AI-PROGNOSIS development cycle, ALTAI’s seven requirements were mapped to six core lifecycle stages, namely: design and specification, data preparation, model development and validation, interface design and deployment, external validation, and cross-cutting governance and workflow (Figure 2). This mapping, also presented in Table S1 in Multimedia Appendix 1, aligns with the high-level expert group on artificial intelligence’s guidance on adapting trustworthiness frameworks to specific system contexts. It enabled the identification of stage-specific trust touchpoints and informed the implementation of safeguards, such as adversarial testing, explainability benchmarking, and privacy-preserving data handling. The integration of these lifecycle stages (Figure 2) is further explored below.

### Lifecycle Integration of ALTAI Requirements

The mapping of ALTAI requirements onto the six lifecycle stages of AI‑PROGNOSIS reflects how trust‑oriented safeguards were embedded as procedural checkpoints rather than post‑hoc evaluations. During design and specification, ALTAI’s requirements for human agency, accountability, and transparency informed early architectural decisions, including the definition of fallback mechanisms, documentation structures, and user‑facing communication strategies. In the data preparation stage, the requirements for privacy, data governance, and fairness guided the establishment of data‑quality routines, access‑control structures, and reviews to identify potential bias proxies. Within model development and validation, ALTAI’s emphasis on technical robustness and safety was operationalized through stress‑testing pipelines, adversarial‑robustness assessments, and explainability benchmarking, ensuring that model behavior was interrogated under realistic perturbations and uncertainty conditions.

As the system moved toward interface design and deployment, requirements related to human oversight, transparency, and diversity were instantiated through iterative co‑design sessions, accessibility reviews, and the integration of explanation elements into the UX and user interface (UI). These activities ensured that model outputs were intelligible, appropriately contextualized, and aligned with user needs. During external validation, ALTAI’s focus on fairness, robustness, and accountability shaped the evaluation of model performance across heterogeneous populations and settings, as well as the refinement of explanation strategies for different stakeholder groups. Finally, in the cross‑cutting governance and workflow stage, ALTAI’s accountability and societal‑ and environmental‑well‑being requirements were reflected in the establishment of ML operations pipelines, lineage tracking, dynamic data‑governance plans, ethics‑board oversight, and monitoring of computational‑resource demands over time, enabling continuous, sustainable monitoring and structured decision‑making throughout the system’s lifecycle. These lifecycle‑specific integrations also set the stage for the more detailed examination of practical, ethical, and organizational tensions that emerged during implementation, which are explored in the following section.

Beyond diagnostic value, the ALTAI adaptation fostered internal reflection among development teams, prompting early-stage deliberation on fallback mechanisms, UI accessibility, and the ethical implications of probabilistic risk scoring. These insights informed both system architecture and stakeholder engagement strategies, reinforcing the role of procedural frameworks as catalysts for trust-aware design. Collectively, these findings emphasize ALTAI’s function not merely as an evaluative instrument, but as a formative scaffold for embedding trust-oriented design logic throughout the development pipeline, as well. This case study exemplifies how procedural frameworks can facilitate the operationalization of ethical and regulatory objectives in clinical AI, with technical teams serving as key intermediaries in translating abstract principles into implementable system architecture.

### Tensions in Pursuit of Trustworthy AI

While procedural frameworks such as ALTAI offer structured guidance for embedding trust into AI development, their translation into clinical practice reveals persistent tensions. These tensions are not solely technical but emerge from the entanglement of ethical, organizational, and regulatory constraints with real-world implementation dynamics. Trust, correspondingly, is not a static attribute in this context, but must be seen as a negotiated, adaptive property shaped and actively maintained across the AI lifecycle by design choices, deployment decisions, and end user interactions [17,31]. Staking out paths to navigate these tensions should therefore be a procedural undertaking, allowing dynamic adaptation on a case-by-case basis, yet offering a guiding rail. Below, we outline key decision-points and how they were navigated in the AI-PROGNOSIS project, using this approach.

### Engineering Clarity Under Complexity

Design-time interventions within AI-PROGNOSIS emphasized risk mitigation through adversarial robustness, constrained model behavior, and transparency mechanisms. Structured data validation pipelines were established to ensure completeness, consistency, and semantic fidelity of clinical inputs. Early-stage vulnerability assessments leveraged tools such as the Adversarial Robustness Toolbox [40] and CleverHans [41] to stress-test model responses under plausible perturbations. These safeguards were necessary but not sufficient, given that trust is also shaped by user perceptions, system intelligibility, and the sociolegal context of deployment.

Explainability was pursued through Shapley additive explanations and local interpretable model-agnostic explanations visualizations [42], integrated into intuitive UX and UI elements, co-developed through co-design sessions with patients, clinicians, and human-computer interaction specialists. The desired output included layout consistency, content hierarchy, accessibility, and performance optimization that can ensure usability across user populations. Additionally, design principles, such as Google’s Material Design or Apple’s Human Interface Guidelines, offered baseline frameworks for further validation through iterative testing with diverse users. These interfaces incorporated risk disclaimers, scenario-specific warnings, and model output rationales, elements shown to foster situational awareness and calibrate expectations [43]. However, tension emerged between model interpretability and predictive fidelity: simpler, more explainable models sometimes underperformed in capturing longitudinal patterns, while high-dimensional neural architecture offered superior accuracy at the cost of intelligibility [44,45].

Transparency obligations are also intersected with institutional and commercial constraints. Open disclosure of model behavior, data lineage, and source code encountered resistance when proprietary intellectual property, reputational risk, or liability exposure were perceived. These experiences echo concerns documented in broader AI governance literature, where explainability is seen as a boundary object, interpreted differently by legal experts, regulators, engineers, and lay users [17,20,46]. Trust, therefore, cannot rely solely on post hoc visualization tools or interface overlays; it must be cultivated through continuous interaction between technical artifacts and epistemic communities.

### Generalization Versus Representativeness

Achieving generalization in clinical AI extends beyond algorithmic optimization. It requires validating performance across subpopulations, health care settings, and temporal shifts, domains where real-world complexity and structural inequities surface. Within AI-PROGNOSIS, data were curated to reflect heterogeneity across age, sex, and disease severity, with feature reviews conducted alongside clinicians to de-risk unintentional bias proxies.

Still, key fairness checks were constrained by unavailable or restricted variables. Under the general data protection regulation [47] and ethical review protocols, the collection of race, ethnicity, and socioeconomic indicators was either prohibited or discouraged, limiting the granularity of bias auditing [9,28,29]. These limitations underscored the tension between privacy-preserving practice and equity-informed auditing. Without disaggregated data, even well-calibrated models can systematically underperform or generate disparate outcomes for minority groups [48]. Moreover, generalizability was not static. Drift monitoring, supported by emerging regulatory requirements such as the EU AI Act’s obligations for continuous post‑market performance surveillance (Articles 12, 14, 15, and 72) [49], is increasingly implemented through dedicated tools like Evidently AI [50]. These enable temporal performance tracking but still require careful configuration to avoid false alarms or blind spots. Participatory workshops were convened with diverse stakeholders to co-define performance thresholds, identify context-specific harms, and design explanation strategies tailored to each stakeholder type [45,51]. In the same vein, external validation ensures that AI systems generalize beyond development data. Explainable AI plays a central role during external validation. Explanation strategies should be adapted to different user groups, from clinicians to patients to engineers. At the same time, co-creation workshops or equivalent co-creation processes (eg, innovation jams, living laboratories, open innovation platforms, lead user collaborations, and crowdsourced design challenges) are valuable for defining these strategies and ensuring a shared understanding of outputs.

These findings suggest that generalization must be reframed: not only as an empirical measure of cross-sample performance, but as a sociotechnical process of aligning predictive behavior with real-world variance, regulatory constraints, and stakeholder expectations. Fairness, interpretability, and robustness cannot be optimized independently; they must be co-engineered through continuous iteration and value-sensitive design [16,52].

### Institutional Scaffolding for Sustainable Trust

Trust, to be enduring, must outlive model deployment. Within AI-PROGNOSIS, governance mechanisms were embedded across the system lifecycle using ML operations pipelines [53] managed via ML flow (an open-source platform for managing ML lifecycle) [54]. These supported reproducibility, lineage tracking, and automated logging of model outputs for compliance and audit purposes. Model documentation, following TRIPOD (Transparent Reporting of a multivariable prediction model for Individual Prognosis or Diagnosis) and Datasheets for Datasets [55] guidelines, facilitates reproducibility and informs external reviewers about data provenance, development conditions, and known limitations. Ethical and legal oversight structures were supported through dynamic data governance plans housed in the European Open Science Cloud’s aggregator for open science [56], which offers version-controlled templates for privacy, security, and access control compliance. An internal ethics board, comprising technical, legal, and clinical representatives, was tasked with monitoring value drift, assessing updates to explainability outputs, and coordinating stakeholder feedback loops.

Sustainability efforts address both environmental impact (eg, energy cost of training pipelines) and downstream clinical implications (eg, deskilling risks or overdependence on AI predictions) [16]. These are increasingly salient concerns as health care systems adopt AI at scale and require not only functional models, but systems that preserve professional autonomy and adapt to evolving sociopolitical contexts [16,52]. Moreover, regulatory compliance, particularly with the general data protection regulation [47], is non-negotiable. Privacy-by-design principles should guide system architecture. Data management teams must ensure ethical oversight, secure processing, and adherence to data-sharing agreements.

Crucially, trust must be institutionally maintained. This requires aligning development workflows with adaptive governance structures capable of incorporating feedback, absorbing policy shifts, and ensuring ethical continuity over time. Static checklists are ill-suited for this role; procedural frameworks, on the other hand, can (and must) evolve into organizational capabilities, rooted in accountability, reflexivity, and stakeholder engagement.

### Technical Focus and Societal Impact

Although the lower importance assigned to societal‑level impacts, for example, effects on work, skills, democracy, or broader social structures (Figures 1 and 2), could be interpreted as a natural consequence of the AI-PROGNOSIS clinical implementation focus, our findings point to a deeper and well‑documented tension in the field of ethical AI. Prior research has shown that technical teams often prioritize system‑proximal requirements (eg, accuracy, safety, and reliability) while viewing societal and macro‑ethical considerations as diffuse, less actionable, or outside their immediate scope of responsibility [57,58]. This divergence does not indicate that societal impacts are irrelevant; rather, it highlights a persistent gap between optimizing AI performance within a clinical workflow and ensuring that technology aligns with broader social, democratic, and labor‑related values. In line with the broader literature on responsible AI, this tension should be understood as an ongoing governance challenge that requires deliberate management rather than acceptance [59,60]. For AI‑PROGNOSIS, this means that even if domain experts perceive societal impacts as less central to immediate development tasks, these considerations must still be systematically integrated into oversight structures, stakeholder engagement processes, and long‑term governance mechanisms. Addressing this misalignment is essential for ensuring that the system evolves not only as a clinically robust tool but also as one that contributes positively to the wider socio‑technical ecosystem in which it will operate.

Taken together, the experience in AI‑PROGNOSIS illustrates how ALTAI can function as a starting scaffold rather than an endpoint for procedural trust. Several trust‑oriented practices emerged organically during development, not because ALTAI prescribed them, but because real‑world clinical, technical, and organizational constraints demanded additional structure. These included the introduction of quantitative lifecycle checks, the systematic integration of stakeholder feedback into design decisions, and the establishment of reproducibility and governance mechanisms that supported continuous oversight. Their emergence demonstrates how ALTAI can trigger deeper procedural adaptations when applied in practice, offering insight into how high‑level principles translate into operational routines within complex clinical AI projects.

### Outlook: Building Living Systems of Trust

This Viewpoint has outlined a procedural approach to embedding trust in clinical AI, grounded in the ALTAI framework [39] and instantiated through the AI-PROGNOSIS project. By integrating ethical, technical, and regulatory safeguards across the AI lifecycle, we have demonstrated how trust can be operationalized not only as a design goal but as a dynamic property of clinical AI systems. Nevertheless, we acknowledge that the survey was limited by its small sample size (n=10) and restriction to AI developers and data scientists from the AI-PROGNOSIS consortium. This effort represents a pilot internal assessment rather than statistically significant or generalizable evidence of ALTAI domain priorities. Moreover, the survey data were collected exclusively from AI developers and data scientists, whereas other key stakeholders, such as people with Parkinson disease, caregivers, and health care professionals, were not included. This may have biased the prioritization of ALTAI subdomains toward the perspectives and priorities of technical experts, which do not necessarily align with end user values and concerns. Involvement of a more diverse and representative group of stakeholders can ensure that domain weighting better reflects end users’ needs and expectations.

Yet, as the field matures, it is increasingly clear that procedural scaffolding alone has its limitations. Trustworthiness must be sustained through adaptive governance, capable of responding to evolving risks, shifting stakeholder expectations, and emerging regulatory mandates. In this regard, the newly introduced European Artificial Intelligence Act (AI Act) [61] represents a pivotal inflection point. Entering into force in August 2024, the AI Act introduces a harmonized, risk-based legal framework for AI across the EU, with specific obligations for high-risk systems, including those deployed in health care [61]. These include requirements for transparency, human oversight, robustness, and postmarket monitoring, many of which align with ALTAI’s procedural ethos but now carry legal enforceability.

Translating ALTAI’s procedural safeguards into the regulatory obligations of the EU AI Act offers a practical pathway for organizations preparing for compliance with high‑risk clinical AI requirements. Several ALTAI mechanisms map directly onto the AI Act’s mandated controls, including lifecycle documentation (eg, data sheets and model cards) that support the AI Act’s technical documentation and record‑keeping duties; structured human‑oversight protocols that align with Articles 14 and 29; risk‑management workflows that mirror the AI Act’s continuous risk‑assessment and mitigation obligations; and data governance practices that reinforce requirements for data quality, representativeness, and bias monitoring. Additionally, ALTAI’s emphasis on fallback planning, robustness testing, and postdeployment monitoring corresponds to the Act’s provisions for postmarket surveillance and performance drift detection. By adopting ALTAI‑like processes early in system design, organizations can establish governance infrastructures that not only enhance trustworthiness but also streamline future conformity assessments under the AI Act.

The AI-PROGNOSIS framework is being continuously adapted to anticipate and respond to these and other regulatory and policy developments. Specifically, future iterations will (1) integrate AI Act compliance checkpoints into development workflows, (2) expand stakeholder engagement to include legal and regulatory experts, and (3) establish mechanisms for continuous postdeployment monitoring and redress. These steps reflect the broader approach, shifting away from principle-based ethics toward procedural trust and institutionalized accountability, where trust is not only designed and cultivated, but governed.

Looking ahead, we argue that trustworthy AI in health care must be conceptualized as a living system, one that evolves through iterative feedback, interdisciplinary and diverse collaboration, and regulatory responsiveness. This requires moving beyond static checklists toward reflexive infrastructures that embed ethical deliberation, stakeholder negotiation, and lifecycle oversight into the core of AI development. As the regulatory landscape crystallizes and clinical adoption accelerates, such infrastructures will be essential to ensure that AI systems remain not only performant but aligned with the values, rights, and expectations of the societies they serve.

## Appendix

Multimedia Appendix 1 Average importance score by ALTAI subgroup and full set of ALTAI checklist responses alongside the corresponding system-generated recommendations.

## Abbreviations

AI: artificial intelligence
ALTAI: Assessment List for Trustworthy Artificial Intelligence
EU: European Union
ISO/IEC TS: International Organization for Standardization/International Electrotechnical Commission Technical Specification
ML: machine learning
PD: Parkinson disease
TRIPOD: Transparent Reporting of a multivariable prediction model for Individual Prognosis or Diagnosis
UI: user interface
UX: user experience

## Data Availability

The datasets generated and analyzed in this study are not publicly available due to access restrictions mandated by the AI‑PROGNOSIS project’s data governance framework. Access may be granted upon reasonable request to the corresponding author and requires prior authorization through the project’s formal data‑access procedures.
