# Supplementary material for: Clinical AI is Not (Yet) Trustworthy-But It Could Be
Source: J Med Internet Res. 2026 Apr 29;28:e85433. doi: 10.2196/85433 (PMC13173088; doi:10.2196/85433)
Supplement: Multimedia Appendix 1 [file jmir_v28i1e85433_app1.docx]

**Viewpoint**

**Clinical AI is not (yet) trustworthy-but it could be**

*Ali Saad^a,†^, Sofia B. Dias^b,†^, Ghada Alhussein^c,d†^, David Lyreskog^e^, Ioannis Gerasimou^f^, Beatriz Alves^c^, Μaarten de Vos^g^, Ioannis Drivas^h^, John Zaras^i^, Andreas Stergioulas^j^, Alex Bensenousi^a^, Leontios J. Hadjileontiadis^d,f,^*✉, Christos Chatzichristos^g,^*✉, and Stelios Hadjidimitriou^f,^*✉, on behalf of the AI-PROGNOSIS Consortium‡*

*^a^AINIGMA Technologies, Leuven, Belgium*

*^b^Interdisciplinary Centre for the Study of Human Performance (CIPER), Faculdade de Motricidade Humana, Universidade de Lisboa, Lisbon, Portugal*

*^c^Faculdade de Motricidade Humana, Universidade de Lisboa, Lisbon, Portugal*

*^d^Department of Biomedical Engineering and Biotechnology, Khalifa University, Abu Dhabi, UAE*

*^e^NEUROSEC, Department of Psychiatry, University of Oxford, Oxford, UK*

*^f^Department of Electrical Computer Engineering, Aristotle University of Thessaloniki, Thessaloniki, Greece*

*^g^Department of Electrical Engineering, STADIUS Center for Dynamical Systems, Signal Processing, and Data Analytics, KU Leuven, Leuven, Belgium*

*^h^Diadikasia Business Consulting Symvouloi Epicheiriseon AE, Athens, Greece.*

*^i^Squaredev, Brussels, Belgium*

*^j^Information Technologies Institute, Centre for Research and Technology Hellas, Thessaloniki, Greece*

*†These authors shared first authorship*

**These authors shared last authorship*

*‡Members are listed at the end of the article*

^✉^*Corresponding authors: Prof. Leontios Hadjileontiadis; Email: leontios@auth.gr, Dr Christos Chatzichristos; Email:* [*christos.chatzichristos@kuleuven.be*](mailto:christos.chatzichristos@kuleuven.be)*, Dr Stelios Hadjidimitriou; Email:* [*shadjidim@ece.auth.gr*](mailto:shadjidim@ece.auth.gr)

Department of Electrical and Computer Engineering

Aristotle University of Thessaloniki

GR 54124 Thessaloniki, Greece

**Appendix**

**Appendix Figure 1: Average importance score by ALTAI subgroup**. ALTAI: Assessment List for Trustworthy Artificial Intelligence.


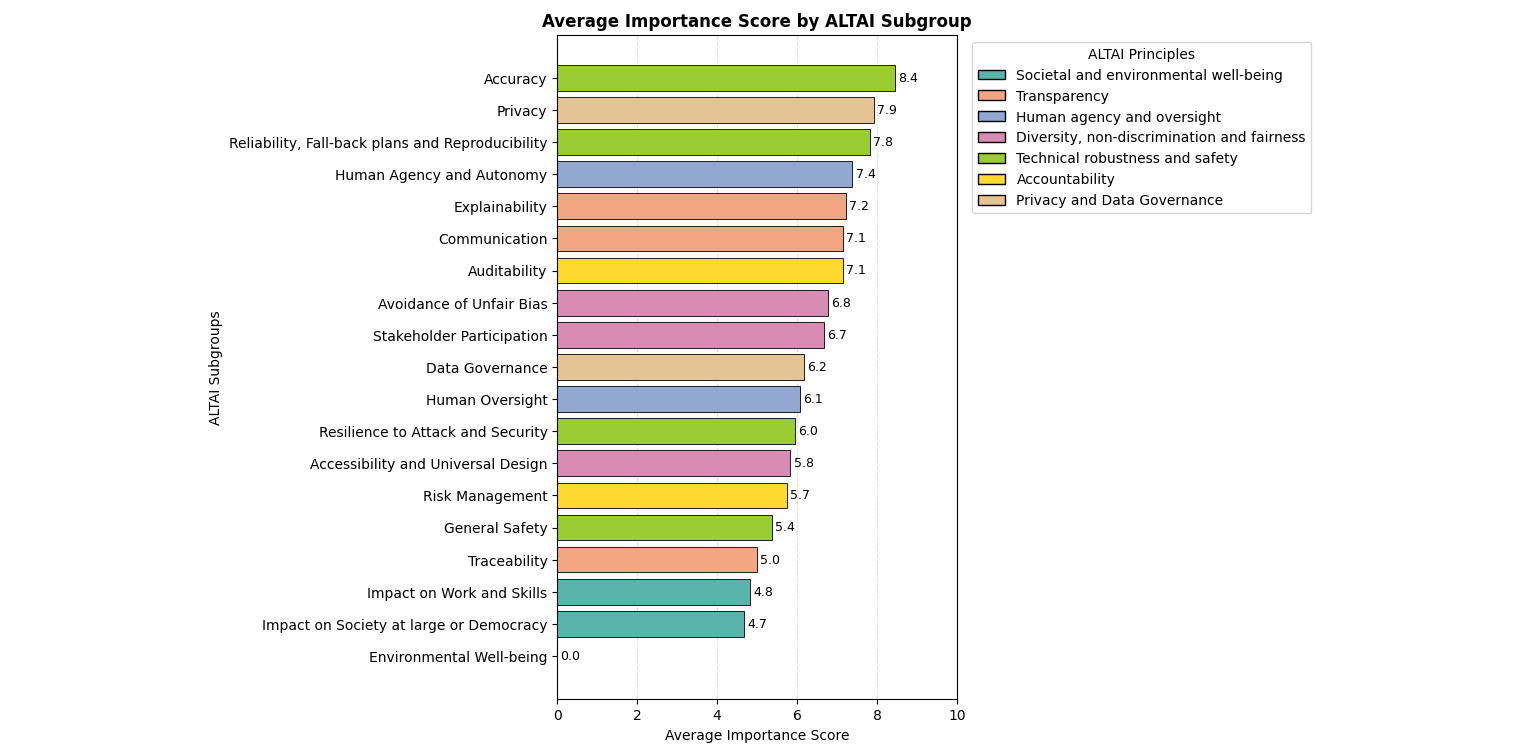


| **Appendix Table S1. Survey questions used to assess the relevance of ALTAI’s seven requirements**. | | |
| --- | --- | --- |
| **ALTAI requirements** | **Relevant to**  **AI-PROGNOSIS**  **[Yes/No]** | **Importance to**  **AI-PROGNOSIS**  **[High/Low/Medium]** |
| **Human agency and oversight** | | |
| Incorporate a process where end-users and/or subjects are adequately made aware that an AI-system influenced the decision, content, advice or outcome. |  |  |
| Ensure that the end-users or subjects are adequately informed that they are interacting with an AI system. |  |  |
| Put in place procedures to avoid that end users over-relying on the AI system. |  |  |
| Put in place any procedure to avoid that the system inadvertently affects human autonomy. |  |  |
| Take measures to deal with the possible negative consequences for end-users or subjects in case they develop attachment. In particular, provide means for the user to have control of the interactions. |  |  |
| Take measures to minimize the risk of addiction by involving experts from other disciplines such as psychology and social work. |  |  |
| Take measures to mitigate the risk of manipulation, including providing clear information about ownership and aims of the system, avoiding unjustified surveillance, and preserving autonomy and mental health of users. |  |  |
| Give specific training to humans (human-in-the-loop, human-on-the-loop, human-in-command) on how to exercise oversight. |  |  |
| Establish detection and response mechanisms in case the AI system generates undesirable adverse effects for the end-user or subject. |  |  |
| Deploy a “stop button” or procedure to safely abort an operation when needed. |  |  |
| Take oversight and control measures to reflect the self-learning or autonomous nature of the AI system |  |  |
| **Technical robustness and safety** | | |
| Assess potential forms of attacks to which the AI system could be vulnerable. |  |  |
| Put in place measures to ensure the integrity, robustness and overall security of the AI system against potential attacks over its lifecycle. |  |  |
| Red-team/pen test the system |  |  |
| Inform users as soon as possible if some new threats are detected. |  |  |
| Define risk, risk metrics and risk levels of the AI system in each specific use case. |  |  |
| Identify the possible threats to the AI system (design faults, technical faults, environmental threats) and the possible resulting consequences. |  |  |
| Assess the risk of possible malicious use, misuse or inappropriate use of the AI system. |  |  |
| Assess the dependency of critical system’s decisions on its stable and reliable behaviour. |  |  |
| Plan fault tolerance via, e.g., a duplicated system or another parallel system (AI-based or “conventional”). |  |  |
| Develop a mechanism to evaluate when the AI system has been changed enough to merit a new review of its technical robustness and safety. |  |  |
| Put in place measures to ensure that the data (including training data) used to develop the AI system is up to date, of high quality, complete and representative of the environment the system will be deployed in. |  |  |
| Put in place a series of steps to monitor and document the AI system’s accuracy. |  |  |
| Consider whether the AI system's operation can invalidate the data or assumptions it was trained on, and how this might lead to adversarial effects (e.g. biased estimators, echo chambers etc.) |  |  |
| Put in place processes to ensure that the level of accuracy of the AI system to be expected by end-users and/or subjects is properly communicated. |  |  |
| Put in place a well-defined process to monitor if the AI system is meeting the goals of the intended applications. |  |  |
| Test whether specific contexts or conditions need to be taken into account to ensure reproducibility. |  |  |
| Put in place verification and validation methods and documentation (e.g., logging) to evaluate and ensure different aspects of the system’s reliability and reproducibility. |  |  |
| Clearly document and operationalize processes for the testing and verification of the reliability and reproducibility of the AI system. |  |  |
| Define tested failsafe fallback plans to address AI system errors of whatever origin and put governance procedures in place to trigger them. |  |  |
| Put in place a proper procedure for handling the cases where the AI system yields results with a low confidence score. |  |  |
| Consider potential negative consequences from the AI system learning novel or unusual methods to score well on its objective function. |  |  |
| **Privacy and Data Governance** | | |
| Take measures to consider the impact of the AI system on the right to privacy, the right to physical, mental and/or moral integrity and the right to data protection. |  |  |
| Consider establishing mechanisms that allow flagging issues related to privacy or data protection concerning the AI system. |  |  |
| When relevant, implement the right to withdraw consent, the right to object and the right to be forgotten in the AI system. |  |  |
| Consider the privacy and data protection implications of data collected, generated or processed over the course of the AI system's lifecycle. |  |  |
| Consider the privacy and data protection implications of the AI system's non-personal training-data or other processed non-personal data. |  |  |
| Whenever possible and relevant, align the AI-system with relevant standards (e.g. ISO, IEEE) or widely adopted protocols for (daily) data management and governance. |  |  |
| **Transparency** | | |
| Consider adopting measures to continuously assess the quality of the input data to the AI system. |  |  |
| Consider adopting adequate logging practices in place to record the decision(s) or recommendation(s) of the AI system |  |  |
| Consider explaining the decision adopted or suggested by the AI system to its end users. |  |  |
| Consider continuously surveying the users to ask them whether they understand the decision(s) of the AI system. |  |  |
| In case of interactive AI system, consider communicating to users that they are interacting with a machine. |  |  |
| Establish mechanisms to inform users about the purpose, criteria and limitations of the decision(s) generated by the AI system |  |  |
| **Diversity, non-discrimination and fairness** | | |
| Consider establishing a strategy or a set of procedures to avoid creating or reinforcing unfair bias in the AI system, both regarding the use of input data as well as for the algorithm design. |  |  |
| Consider diversity and representativeness of end-users and/or subjects in the data. |  |  |
| Test for specific target groups or problematic use cases. |  |  |
| Research and use publicly available technical tools, that are state-of-the-art, to improve your understanding of the data, model and performance. |  |  |
| Assess and put in place processes to test and monitor potential biases during the entire lifecycle of the AI system (e.g. biases due to possible limitations stemming from the composition of the used data sets (lack of diversity, non-representativeness). |  |  |
| Consider diversity and representativeness of end-users and/or subjects in the data. |  |  |
| Put in place educational and awareness initiatives to help AI designers and AI developers be more aware of the possible bias they can inject in designing and developing the AI system. |  |  |
| Depending on the use case, ensure a mechanism that allows for the flagging of issues related to bias, discrimination or poor performance of the AI system. |  |  |
| You should establish clear steps and ways of communicating on how and to whom such issues can be raised. |  |  |
| Identify the subjects that could potentially be (in)directly affected by the AI system, in addition to the (end)-users. |  |  |
| Your definition of fairness should be commonly used and should be implemented in any phase of the process of setting up the AI system. |  |  |
| Consider other definitions of fairness before choosing one. |  |  |
| Consult with the impacted communities about the correct definition of fairness, such as representatives of elderly persons or persons with disabilities. |  |  |
| Ensure a quantitative analysis or metrics to measure and test the applied definition of fairness. |  |  |
| Establish mechanisms to ensure fairness in your AI system. |  |  |
| You should ensure that the AI system corresponds to the variety of preferences and abilities in society. |  |  |
| You should assess whether the AI system's user interface is usable by those with special needs or disabilities or those at risk of exclusion. |  |  |
| You should ensure that Universal Design principles are taken into account during every step of the planning and development process, if applicable. |  |  |
| You should take the impact of the AI system on the potential end-users and/or subjects into account. |  |  |
| You should assess whether the team involved in building the AI system engaged with the possible target end-users and/or subjects. |  |  |
| You should assess whether there could be groups who might be disproportionately affected by the outcomes of the system. |  |  |
| You should assess the risk of the possible unfairness of the system onto the end-user's or subject's communities. |  |  |
| You should consider a mechanism to include the participation of the widest range of possible stakeholders in the AI system’s design and development. |  |  |
| **Societal and environmental well-being** | | |
| Consider the potential positive and negative impacts of your AI system on the environment and establish mechanisms to evaluate this impact. |  |  |
| Define measures to reduce the environmental impact of your AI system’s lifecycle and participate in competitions for the development of AI solutions that tackle this problem. |  |  |
| Inform and consult with the impacted workers and their representatives but also involve other stakeholders. Implement communication, education, and training at operational and management level. |  |  |
| Take measures to ensure that the work impacts of the AI system are well understood on the basis of an analysis of the work processes and the whole socio-technical system. |  |  |
| Take measures to counteract de-skilling by means of continuous training, especially in areas sensitive in terms of safety and security. |  |  |
| Provide training opportunities and materials for re- and up-skilling measures. |  |  |
| Assess the societal impact of the AI system’s use beyond the (end-)user and subject, such as potentially indirectly affected stakeholders or society at large. |  |  |
| Take actions to minimize potential societal harm of the AI system. |  |  |
| Take measures that ensure that the AI system does not negatively impact democracy. |  |  |
| **Accountability** | | |
| Establish mechanisms that facilitate the AI system’s auditability (e.g. traceability of the development process, the sourcing of training data and the logging of the AI system’s processes, outcomes, positive and negative impact). |  |  |
| Ensure that the AI system can be audited by independent third parties. |  |  |
| Foresee any kind of external guidance or third-party auditing processes to oversee ethical concerns and accountability measures. |  |  |
| Organise risk training for developers and deployers to inform them about the potential legal framework applicable to the AI system. |  |  |
| Establishing an AI ethics review board or a similar mechanism to discuss the overall accountability and ethics practices, including potential unclear grey areas. |  |  |
| Establish a process to discuss and continuously monitor and assess the AI system's adherence to this Assessment List for Trustworthy AI (ALTAI). |  |  |
| Establish a process for third parties (e.g. suppliers, end-users, subjects, distributors/vendors or workers) to report potential vulnerabilities, risks or biases in the AI system. |  |  |
| Ensure that redress-by-design mechanisms are put in place for applications that can adversely affect individuals. |  |  |

| **Appendix Table S2. Detailed ALTAI Self-Assessment and Prioritization for AI-PROGNOSIS**. | | |
| --- | --- | --- |
| **AI cycle** | **ALTAI requirement** | **Summary of relevant components related to each AI cycle stage** |
| **Design and specification** | **HAO** | Implementation of protocols avoiding over-reliance by end users. Establishing processes to prevent inadvertent effect of the AI system on human autonomy. Enabling human oversight human-in-the-loop, human-on-the-loop, and human-in-command). |
|  | **TRS** | Definition of risk metrics and levels. Establishing procedures for monitoring and documenting the system's accuracy, ensuring clear communication of expected accuracy to end-users or subjects. |
|  | **PDG** | Consider the impact of the AI system on the right to privacy, the right to physical, mental and/or moral integrity and the right to data protection. Align the AI-system with relevant standards or protocol for data management and governance. |
|  | **TPR** | Identification of user needs regarding explainability of the AI system’s decision(s). |
|  | **DnDF** | Utilization of publicly available technical tools to enhance the understanding of data, model, and performance. Identification of potential biases throughout the AI system's lifecycle. Definition of group/individual fairness goals and of protected attributes. Establish mechanisms for flagging bias, discrimination, or low performance issues. |
|  | **ACC** | Enabling and facilitating the AI system’s auditability. |
| **Data preparation** | **TRS** | Ensuring the high quality of the data. |
|  | **PDG** | Application of data privacy mechanisms for data collected, generated or processed over the course of the AI system's lifecycle. |
|  | **DnDF** | Avoid, correct, and monitor unfair bias. |
| **Development and internal validation** | **TRS** | Assessing potential vulnerabilities of the AI system. Implementing robust measures throughout the system's lifecycle to ensure its robustness and overall security. Ensuring the reproducibility of the developed AI models. Performance benchmarking, including reliability assessment through uncertainty and generalisation evaluation. |
|  | **PDG** | Model assessment in terms of data leaking, missing data, and membership/attribute inference vulnerabilities. |
|  | **TPR** | Explanation of the decision by the AI system to its end users (explainability benchmarking). |
|  | **DnDF** | Assessing and mitigating bias throughout the model development phase. |
| **UX/UI and deployment** | **HAO** | Ensuring end-users or subjects are informed about the AI system's influence on decisions or outcomes. Establishing mechanisms to detect and address any adverse effects generated by the AI system on end-users or subjects (Adversarial testing in early deployment). |
|  | **TRS** | Notification of users upon detecting new threats. Evaluation of the dependency of critical system decisions on its stable behaviour. |
|  | **PDG** | Enabling end-users or subject to have the right to withdraw consent, object, and be forgotten in the AI system. |
|  | **TPR** | Ensuring that the end-users understand the decision(s) of the AI system. |
|  | **DnDF** | Assessing the system's user interface for usability by individuals with special needs, disabilities, or those at risk of exclusion is essential. |
| **External validation** | **TRS** | Performance evaluation, including reproducibility and reliability evaluation. |
|  | **TPR** | Evaluation of quality and adequacy of model output explanations by end-users in clinical studies. |
|  | **DnDF** | Diverse participant sample in clinical studies for external validation of performance and user acceptance evaluation. |
| **Overarching management and workflow** | **TRS** | Assessment of the risk of possible malicious use, misuse or inappropriate use of the AI system. |
|  | **PDG** | Supporting AI governance strategies. Establishing mechanisms to flag issues related to privacy or data protection. |
|  | **SEW** | Ensure the understanding and assessment of the impacts of the AI system on work, skills, and society at large as part of the general socio-ethical implications assessment. |
| ALTAI: Assessment List for Trustworthy Artificial Intelligence; HAO: Human agency and oversight; TRS: Technical robustness and safety; PDG: Privacy and data governance TPR: Transparency; DnDF: Diversity, non-discrimination and fairness; SEW: Societal and environmental wellbeing; ACC: Accountability | | |
